# Wearable Capacitive Tactile Sensor Based on Porous Dielectric Composite of Polyurethane and Silver Nanowire

**DOI:** 10.3390/polym15183816

**Published:** 2023-09-19

**Authors:** Gen-Wen Hsieh, Chih-Yang Chien

**Affiliations:** 1 Institute of Lighting and Energy Photonics, College of Photonics, National Yang Ming Chiao Tung University, 301, Gaofa 3rd Road, Section 2, Guiren District, Tainan 71150, Taiwan; 2 Institute of Photonic System, College of Photonics, National Yang Ming Chiao Tung University, 301, Section 2, Gaofa 3rd Road, Guiren District, Tainan 71150, Taiwan; d0149574@gmail.com

**Keywords:** capacitive tactile sensor, dielectric composite, porous polyurethane, silver nanowire, wearable electronic

## Abstract

In recent years, the implementation of wearable and biocompatible tactile sensing elements with sufficient response into healthcare, medical detection, and electronic skin/amputee prosthetics has been an intriguing but challenging quest. Here, we propose a flexible all-polyurethane capacitive tactile sensor that utilizes a salt crystal-templated porous elastomeric framework filling with silver nanowire as the composite dielectric material, sandwiched by a set of polyurethane films covering silver nanowire networks as electrodes. With the aids of these cubic air pores and conducting nanowires, the fabricated capacitive tactile sensor provides pronounced enhancement of both sensor compressibility and effective relative dielectric permittivity across a broad pressure regime (from a few Pa to tens of thousands of Pa). The fabricated silver nanowire–porous polyurethane sensor presents a sensitivity improvement of up to 4−60 times as compared to a flat polyurethane device. An ultrasmall external stimulus as light as 3 mg, equivalent to an applied pressure of ∼0.3 Pa, can also be clearly recognized. Our all-polyurethane capacitive tactile sensor based on a porous dielectric framework hybrid with conducting nanowire reveals versatile potential applications in physiological activity detection, arterial pulse monitoring, and spatial pressure distribution, paving the way for wearable electronics and artificial skin.

## 1. Introduction

Flexible tactile sensors that can convert stimuli into electronic signals have garnered significant attention for their potential use in the development of electronic skin, healthcare monitoring, and human–machine interaction. These tactile sensors can generally be classified into several physical transduction mechanisms, including piezoresistive [1], capacitive [2,3,4], piezoelectric [5,6,7], triboelectric [8,9,10], and photo-responsive [11,12]. Among them, capacitive tactile sensors possessing elastomeric dielectric rubbers/polymers have been intensively studied owing to their simple geometry, superior flexibility, inexpensive material cost, and low power consumption. However, the sensing ability of these elastomer-based capacitive tactile sensors is restricted by their compressibility. Upon a small amount of applied pressure, these elastomers cannot produce enough deformation. Additionally, after the removal of pressure, the time required for restoration to their initial status is excessively long due to the viscoelastic property of the unstructured films.

Although several approaches involving the introduction of geometric microstructures or interior porous networks into the dielectric layers have been broadly investigated [2,3,13,14,15,16], such efforts can only be valuable in low-pressure regimes. Upon application of a medium-to-large amount of pressure, these gaps or pores can be almost completely squeezed, and the flattened film becomes hardly compressible. Moreover, incorporating nanofillers, such as MXene [17,18], metals/metal oxides [19,20,21,22,23], or carbons [24,25], into polymer dielectrics would likely enhance the relative dielectric permittivity and compressibility. Yet, high loading of zero- or two-dimensional nanofillers incorporated into the polymer host may generate uneven dispersion and aggregation, thus deteriorating their flexibility and processability.

For the above reasons, finding an augmentative means by comprehensively combining two or more aspects is intriguing for high performance capacitive tactile sensing. Park et al. [26] developed a stretchable energy-harvesting and tactile-sensing electronic sensor by using porous PDMS and microstructured PDMS with air gaps as the dielectric layers. The air gap increased sensitivity in the low-pressure regime, and the porous PDMS extended capacitance response in a wider-pressure regime. Further, a sophisticated capacitive pressure sensor utilizing a porous pyramid dielectric film was developed by Yang et al. [27], which presented an unprecedented sensitivity with respect to that of the solid pyramid layer. 

In addition to the combination of porous network with surface microstructure, the microstructured or porous elastomer can possibly be made into a hybrid with nanostructures to improve sensor sensitivity. Rana et al. [28] created a composite dielectric comprising ceramic BaTiO_3_ nanoparticles and Ecoflex polymer with a double-stage microstructure that can improve sensor compressibility and effective relative dielectric permittivity under pressure. Mu et al. [29] also demonstrated a porous PDMS matrix hybrid with ceramic CaCuTi_4_O_12_ nanoparticles, revealing beneficial compressibility and sensitivity over that of flat dielectric materials. Moreover, Wen et al. [30] proposed a porous Ecoflex with multiwalled carbon nanotubes. When the air pores were fully squeezed, the resulted relative dielectric permittivity of the composite was significantly improved by the incorporated nanotubes. Guo et al. [31] selected a series of carbon nanotubes with different aspect ratios as nanofillers for the microstructured PDMS dielectric film, by which, the sensitivity could be adjusted accordingly. Pruvost et al. [32] deposited carbon black onto the inner surface of porous PDMS, presenting enhanced effective dielectric permittivity and relative capacitance change upon applying pressure. Overall, these demonstrations have prompted the pursuit of a simple and cost-effective approach to the development of a wearable and biocompatible dielectric composite with high performance capacitive tactile sensing. 

Here, we present a composite dielectric material in which one-dimensional silver nanowire is combined with porous elastomeric polyurethane for the development of wearable capacitive tactile sensors. The former, holding unique conducting and dielectric polarization properties, has received widespread attention for optoelectronic, sensing, and energy harvesting applications; the latter has recently been adopted as a biomimetic membrane for artificial skin because of its good dimensional stability, air permeability, bio-safe property, and low-cost processability. The proposed polyurethane-based nanocomposite with open porosity is formed by a salt crystal-templated pore-generation process and blended with high aspect ratio silver nanowires with different loadings. The fabricated silver nanowire–porous polyurethane sensor can exhibit superior tactile sensing capability where a minimum detectable pressure change of ~0.3 Pa and a broad sensing range of up to 20 kPa are demonstrated. This capacitive tactile sensing device also displays the versatility required for use in various applications, such as human motion detection, health monitoring, spatial recognition, and electronic skin.

## 2. Experimental

### 2.1. Preparation of Silver Nanowires

Silver nanowires (AgNWs) were synthesized by a modified polyol process [33,34,35]. Ethylene glycol (10 mL, Sigma-Aldrich, St. Louis, MO, USA), poly(vinylpyrrolidone) (0.12 g, Sigma-Aldrich), and silver nitrate (AgNO_3_, 0.18 g, Sigma-Aldrich) were thoroughly mixed in a flask until the solution turned clear light-yellow; then, a ferric chloride solution (220 μL, 11.2 mM, Sigma-Aldrich) was promptly injected into the solution and vigorously stirred (~90 s) to bring about the formation of Ag nucleation seeds. The mixture was then placed in an oven (135 °C for 5 h) for cultivating AgNWs (note: opaque grey solution may likely imply the existence of long nanowires). Afterward, the growth was terminated by placing the resultant solution into a water bath at ambient temperature. To remove the undesired residues, the solution was then washed and centrifugated with acetone (at 5000 rpm for 5 min). The sediment of AgNWs was further purified in ethanol and centrifuged using this process for three repetitions. Finally, the AgNWs could be stored as dispersion in ethanol or acetone for the following fabrication.

### 2.2. Preparation of Silver Nanowire–Porous Polyurethane Capacitive Tactile Sensors

For producing PU-based elastomeric composite dielectric materials, the AgNW dispersion in acetone was firstly mixed with the diisocyanate curing agent (Clear FlexTM 30-Part B, Smooth-On) by magnetic stirring. The mixture was then degassed and dried in a vacuum desiccator at 60 °C for 30 min to remove the gas and acetone, resulting in randomly dispersed AgNWs in Part B (note: the amount of AgNWs can thus be estimated by the weight difference of Part B with and without nanowire). Therefore, a viscous solution of a clear polyol-based urethane liquid compound (Clear FlexTM 30-Part A, Smooth-On) and the diisocyanate curing agent (at a weight ratio of 1:0.94 for polymerization) with Ag nanowires of different weight ratios (0, 1.0, 2.0, and 3.0 wt %, respectively) was blended by a mechanical mixer (1000 rpm, 30 min) to reach a thorough and even dispersion. The hybrid solution was then mixed with a certain amount of pre-sieved NaCl crystals (~100–150 μm, 150–200 μm, or 200–250 μm in size, Sigma-Aldrich) thoroughly. Afterwards, the mixture was poured into a mold (height: 450 μm, covered with a seal) and cured at 60 °C for 5 h. Subsequently, the embedded NaCl was leached out by immersing in water (90 °C for 72 h, with changing the water every 12 h) in order to form well-dispersed microcavities in the PU film. After drying at 100 °C for 2 h, a AgNW–porous PU elastomeric film was obtained. The resulting dielectric film was peeled off, which could be cut into various sample sizes. Pristine porous PU or flat PU composite (without nanowire or NaCl template) were prepared in a similar manner.

To produce elastomeric electrodes, the AgNW dispersion in ethanol (~0.5 mg mL^−1^) was spray-coated by a N_2_-driven air spray gun (GYD-1000MDSC, Gison Pneumatic Tools, Taichung, Taiwan) onto the flat PU films through a shadow mask; the spray pressure and distance were controlled at 2 bar and 5 cm, respectively. The films were heated on a hotplate (60 °C for 20 min) to ensure that the ethanol could evaporate rapidly. The sheet resistance of the resultant AgNW electrodes was around 1–5 Ω sq^−1^. Note that the nanowire solution was pre-sieved by a 200 μm wire mesh in order to avoid clogging at the nozzle of the spray gun. Finally, both sides of the prepared AgNW–porous PU dielectric composite were sandwiched between two AgNW/PU electrodes using a thin adhesion layer of thermal-cured PU. Copper wires were used to connect the impedance analyzer to the fabricated sensors.

### 2.3. Characterization and Measurement

The morphology, crystalline structure, and composition of the Ag nanowires were characterized by scanning electron microscopy (SEM, SU8000 FESEM, HITACHI, Tegama, Japan) and high-resolution transmission electron microscopy (HRTEM, JEM-2100F CS STEM, JEOL, Akishima, Japan) with selected area electron diffraction and energy dispersive X-ray spectroscopy. The cross-sectional morphology of flat PU, porous PU, and Ag nanowire–porous PU nanocomposites was analyzed by scanning electron microscopy (SEM, SU8000 FESEM, HITACHI, Tegama, Japan). Capacitance vs. pressure loading measurements were carried out by Agilent E4980AL Precision Impedance Analyze (at 15 kHz frequency with a.c. bias of 2.0 V, Keysight, Santa Rosa, CA, USA). The relative dielectric permittivity of the PU-based films was measured using a dielectric test fixture (Agilent 16451B, Keysight, Santa Rosa, CA, USA). The tactile sensing performance for the Ag nanowire–porous PU were presented alongside those of flat PU and porous PU devices. All experiments were performed at ambient environment.

To apply the proposed sensor for real-time wireless measurement, a data acquisition (DAQ) circuit board was established by a desktop PCB printer (V-One, Voltera, Waterloo, ON, Canada), which assembled a capacitance to digital convertor (AD7151, Analog Devices, Wilmington, MA, USA), a 32-bit microprocessor (Arduino Pro Min 328, SparkFun, Niwot, CO, USA) and a Bluetooth module (HC-06, Olimex Ltd., Plovdiv, Bulgaria). The DAQ board can acquire and convert the capacitance value from the proposed AgNW–porous PU sensor into a digital value, and can provide wireless transduction between the circuit board and a mobile phone.

## 3. Results and Discussion

**Figure 1:**
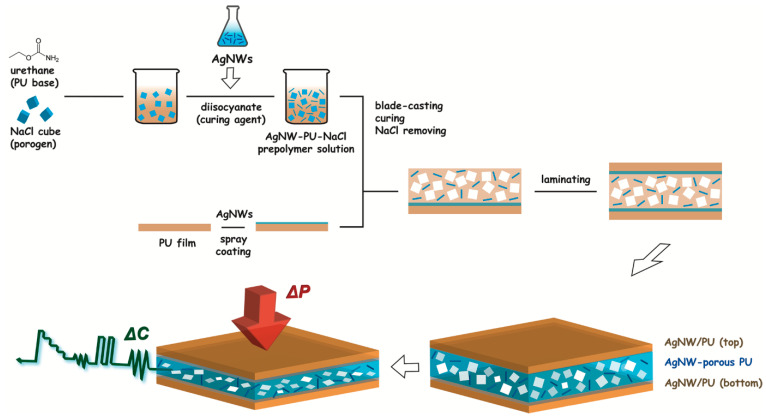
Schematic illustration for fabricating a wearable and biocompatible polyurethane-based capacitive tactile sensor that incorporates NaCl-templated cubic pore and silver nanowire as enablers for external stimuli sensing.

**Figure 2:**
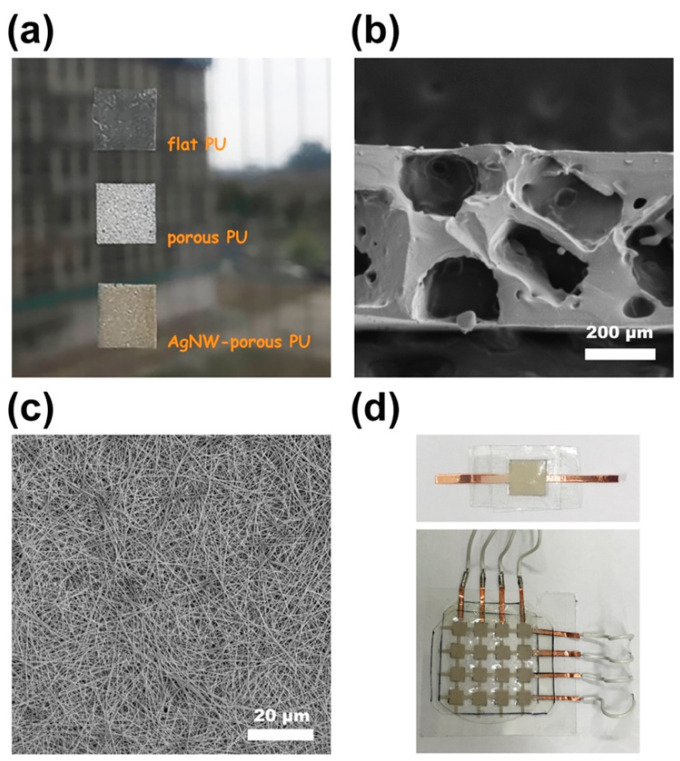
(**a**) Optical image of the flat PU, porous PU, and AgNW–porous PU dielectric composites. (**b**) Cross-sectional SEM image of a NaCl-templated porous PU film. (**c**) SEM image of the incorporated Ag nanowires. (**d**) Photo images of the fabricated AgNW–porous PU capacitive tactile sensors: a single cell and a multipixel array.

The proposed wearable and biocompatible PU-based tactile sensor was made of a sandwich-type capacitor layout (see the schematic diagram in Figure 1) in which the intermediate porous dielectric composite was formulated by incorporating NaCl-templated cubic pores and AgNWs into the PU matrix. A batch of the dielectric composites with varying pore sizes and/or nanowire loadings was fabricated and characterized along with their capability for tactile sensing. The optical and SEM images of the PU-based composite films are presented in Figure 2a,b. The thickness of each composite film was ~450 μm; the diameter and length of the incorporated AgNWs were ~200 nm and ~80–100 μm, respectively (Figure 2c and Figure S1). Then, each type of dielectric film, including AgNW−porous PU, porous PU, or flat PU, was sandwiched between the top and bottom AgNW/PU electrodes accordingly. The photo images of the fabricated capacitive tactile sensors are also presented in Figure 2d.

First, we assessed the size impact of NaCl-templated pores on the capacitor characteristics of the dielectric films. The lateral sizes of NaCl cubic crystals in the range of 100–150, 150–200, to 200–250 μm were categorized by sieving cooking sea salt crystals through different stainless-steel wire meshes (see Figure S2a–c). The weight ratio of the NaCl crystals to be mixed into the PU matrix was 50%, which could ensure the complete removal of the involved crystals as well as the smooth formation of the porous PU films with open pores (Figure 2b). The SEM-EDS spectrum (Figure S3) reveals that the compositional elements of PU such as carbon and oxygen (with deposited Au for observation) exist. Additionally, evidently, no Na or Cl from the salt crystals remained inside. When an external vertical load was applied, the distance (*d*) between the two electrodes as well as the relative dielectric permittivity (*ε_r_*) of these porous PU capacitors were both changed due to the pressure-induced compression. To ensure that a uniform pressure load would be applied, we employed a precision micropipette to apply water droplets (20 μL for each press, ∼20 ± 0.5 mg, corresponding to a pressure load of ∼2.0 Pa) into a small open cube that was firmly fixed on the sensor surface (area: 10 mm × 10 mm). The measured capacitance value of each sensor is referred as *C_0_* (without pressure load) and *C* (with pressure load), respectively. The capacitance of the device and the relative change in capacitance can be defined as [36],

(1) $$C=\frac{\varepsilon_{0}\varepsilon_{r}A}{d}$$

(2) $$\frac{∆C}{C_{0}}=\frac{C-C_{0}\;}{C_{0}}=\frac{\varepsilon_{0}\varepsilon_{r}\;(A/d)}{\varepsilon_{0}\varepsilon_{r0}\;(A/d_{0})}-1=\frac{\varepsilon_{r}}{\varepsilon_{r0}}\cdot \frac{d}{d_{0}}-1$$

where $\varepsilon_{0}$ and A are the permittivity of the vacuum and the area of the overlapped electrodes, respectively; ΔC represents the capacitance variation between C_0_ and C; d_0_ and d represent the distance between electrodes without and with pressure load, and ε_r0_ and ε_r_ represent the relative dielectric permittivity without and with pressure load, respectively. As can be seen in Figure S4, each data set presents how the capacitive tactile sensor responds to applied pressure. The larger the NaCl-templated pores involved, the higher the capacitance responses of the porous PU film, due to the larger geometric deformation of the dielectric film under pressure. Hence, we selected NaCl cubic crystals in the range of ~200–250 μm as the porogens for the proposed capacitive tactile sensors for the following study. 

**Figure 3:**
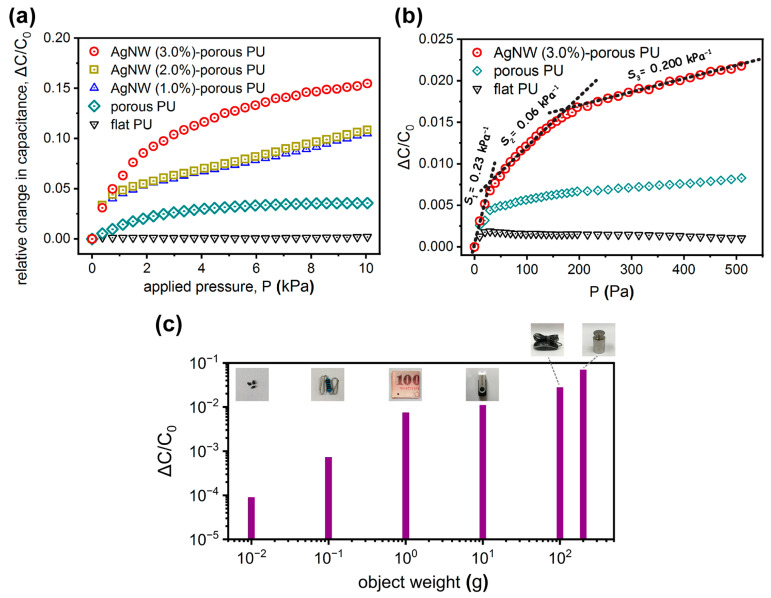
(**a**) Measured relative capacitance change (Δ*C/C_0_*) as a function of the applied pressure (*P*) for the capacitive pressure sensors with different types of dielectric layers: flat PU, porous PU, and AgNW–porous PU (loading: 1.0, 2.0, and 3.0 wt%). (**b**) Magnified pressure–response plots for a flat PU, a porous PU, and a AgNW (3.0 wt%)–porous PU in the pressure range of 0–500 Pa. (**c**) Dynamic responses of a AgNW–porous PU capacitive tactile sensor bearing different objects ranging from ~10 mg to 200 g.

We thereafter proceeded with an investigation of the fabricated capacitive tactile sensors with different dielectric layers, i.e., flat PU, porous PU, and AgNW–porous PU (nanowire loading: 1.0, 2.0, and 3.0 wt%). The relative change in capacitance (ΔC/C_0_) as a function of the applied pressure (P) for these PU-based sensors is presented in Figure 3a. Generally, these devices all resembled a quick ascending shift with low applied pressure (0–500 Pa) and a rather slight increment with high applied pressure (500 Pa–10 kPa), which was commonly associated with the elastic and dielectric properties of polymeric-based dielectric films [16]. For the flat PU sensors, the capacitance change was simply a function of the reduced dielectric thickness since its relative dielectric permittivity (ε_r,flat_ = ε_r0,flat_ = 7.95) remained as a constant (see Figure S5a). With small applied pressure, the ΔC/C_0_ of the flat PU sensors increased linearly because of its elastic deformation; under moderate-to-large pressure, the value was nearly saturated regarding the hardly compressed dielectric. Furthermore, for the porous PU sensors (ε_r0,pore_ = 5.03), the presence of NaCl-templated cubic air pores in the PU matrix could make the dielectric layer softer, thus inducing a larger deformation due to the improved compressibility of the porous layer (Figure S5b). Meanwhile, during compression, the air pores (with relative low relative dielectric permittivity, ε_r,air_ = 1) could be more easily compressed and then replaced by the PU matrix (with relative high relative dielectric permittivity). These could result in dielectric thickness reduction and an effective increase in relative dielectric permittivity (Δε_r,pore_). However, with a high-pressure load, these pores could be fully squeezed; the relative dielectric permittivity of the porous film could thus eventually reach a saturated status, similar to the value of stressed flat PU. Specifically, these AgNW–porous PU capacitive tactile sensors presented superior capacitance response with respect to those flat PU and porous PU devices, as can be seen in Figure 3a. The nanowire loading in the porous dielectric layer can lead to different sensing capabilities; specifically, the relative change in capacitance of the AgNW (3.0 wt%)–porous PU composite was the most pronounced (ε_r0,pore+AgNW_ = 5.42). During compressing, we anticipated that the volumetric change of the porous cubic structure along with the involved high dielectric nanowires in the dielectric network might provide a beneficial buffer effect and an effective increase in relative dielectric permittivity. The capacitance response can be determined by comprehensive consideration of the mechanical strength of the dielectric material and the variation in relative dielectric permittivity (Δε_r,pore+AgNW_) during vertical compression (Figure S5c). Therefore, under applied pressure, the effective relative dielectric permittivity of the AgNW–porous PU composite layer is not only affected by the concentration of nanofillers, but is also influenced by the compression behavior. However, we notice that excessive loading of nanowires has a more negative impact on the capacitance response because the increased mechanical strength of the composite film can suppress the desired effect of dielectric permittivity. Careful optimization of the sensor geometry, dielectric and physical properties, and the interfacial interactions between nanowires and polymer host are critical to maximize tactile sensing capability. 

Moreover, the selected magnified data of the AgNW (3.0 wt%)–porous PU sensor, along with the flat PU and the porous PU at the regime of 0–500 Pa, are compared in Figure 3b, from which we have extracted each slope as the corresponding pressure sensitivity (S), defined as [36],

(3) $$S=\frac{∆C/C_{0}}{∆P}=\frac{\frac{\varepsilon_{r}}{\varepsilon_{r0}}\cdot \frac{d}{d_{0}}-1}{∆P}$$

where Δ*C* represents the capacitance variation, *C*_0_ is the capacitance without pressure load, and Δ*P* is the applied pressure variation, $\varepsilon_{r0}$ and $\varepsilon_{r}$, and *d*_0_ and *d* represent the relative dielectric permittivity and the relative distance between the electrodes, without and with pressure load, respectively. The greater *S* value indicates that the capacitor is more capable of sensing the pressure change. When the applied pressure was 0–30 Pa, the sensitivities of the flat PU and the porous PU were found to be 0.06 and 0.14 kPa^−1^, respectively. On the other hand, the sensitivity of the AgNW (3.0 wt%)–porous PU capacitive tactile sensor greatly increased to 0.23 kPa^−1^, which was ~3.8 times that of the flat PU sensor and ~1.6 times over that of the porous PU device. In the regime of 30–200 Pa, the sensitivity of those porous PU and flat PU were greatly reduced (0.012 and <0.001 kPa^−1^, respectively). In contrast, the AgNW–porous PU sensor still remained at the value of 0.06 kPa^−1^, which was ~5 times that of the porous PU sensor and ~60 times over that of the flat PU device. For a broader sensing regime (i.e., 2–10 kPa, see Figure 3a), the pressure sensitivities of AgNW (3 wt%)–porous PU, porous PU, and flat PU capacitors were 0.009, 0.002, and <0.001 kPa^−1^, accordingly. Thereafter, we can simply employ our tactile sensor to perceive a variety of items by using the same sensor, such as seeds, a resistor, a piece of paper cash, a USB flash drive, a computer mouse, or a standard metal weight, equal to the external pressure load ranging from 1.0 Pa to 20 kPa (see Figure 3c). Overall, our proposed AgNW–porous PU capacitive tactile sensor can present reasonable response over a wide pressure regime.

**Figure 4:**
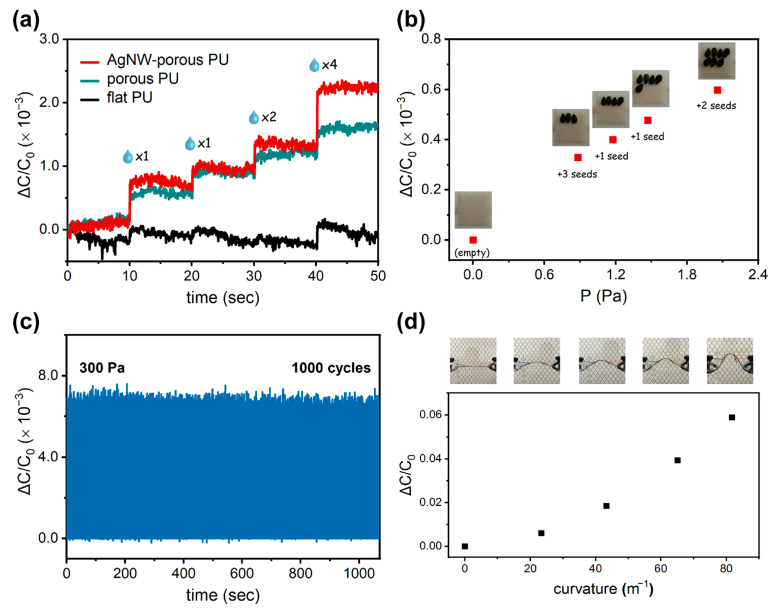
Minimum detectable pressure test for the PU-based capacitive tactile sensors by means of the sequential detection of (**a**) water droplets and (**b**) sesame seeds, respectively. (**c**) Durability test of a AgNW–porous PU film for 1000 cycles at 300 Pa. (**d**) Relative capacitive change as a function of bending curvature from 0 to 80 m^–1^. The insets show the photographs of the test device in different bending states.

Moreover, we examined the minimum detectable pressure (the limit of detection) for the proposed AgNW–porous PU sensor, for which a tiny amount of water droplets (~10 mg per drop) were applied onto the device by using a precision micropipette. As can be seen in Figure 4a, the increments of capacitance change were clearly discerned regarding the subtle addition of 1.0 Pa (one drop), 2.0 Pa (two drops), 2.0 Pa (two drops), and 4.0 Pa (four drops), successively. For comparison, the flat PU device only presented an obscure response. Additionally, we further placed a few sets of sesame seeds one after another, such as three seeds, one seed, one seed, and two seeds, on a thin PET sheet covering the entire sensing area of the sensor. While each set was placed, the device transduced the subtle variation into a capacitance signal (Figure 4b). This equaled an ultrafine response as low as only around 0.3 Pa due to the load of just one sesame seed (~3 mg). We remarked that this tiny value represented an important parameter in the acquisition of any ultralow load. This is also comparable with the minimum detectable pressure achieved in recent published works, mostly with sophisticated design and complex fabrication: 0.1 Pa [3,27,37], 1 Pa [22], 5 Pa [38], 9 Pa [32], 15 Pa [39], 20 Pa [21]. The features of recently developed capacitive tactile sensor technologies, in terms of device components, maximum sensitivity, limit of detection, response time, and operation pressure range, are also summarized in Table S1.

In addition, we observed the cyclic loading–unloading behavior of the AgNW–porous PU capacitive tactile sensor over 1000 times (applied pressure: 300 Pa; frequency: 0.5 Hz). As shown in Figure 4c, the device presented a steady and reliable sensing capability in which the capacitance varied rapidly during pressure load and unload. The response time and recovery time were as short as 100 ms and 100 ms, respectively, which can be very useful in dynamic and real-time pressure monitoring. Moreover, the bending response of our fabricated sensor was further characterized, which was carried out by affixing a pair of metal clamps to the device (one fixed and the other movable, driven by a linear motor). The relative capacitance change increased gradually as the bending curvature increased from 0 to 80 m^–1^ (Figure 4d), which was likely due to the reduced gap between the parallel electrodes. This implies that our PU-based tactile sensor has remarkable flexibility and mechanical stability.

**Figure 5:**
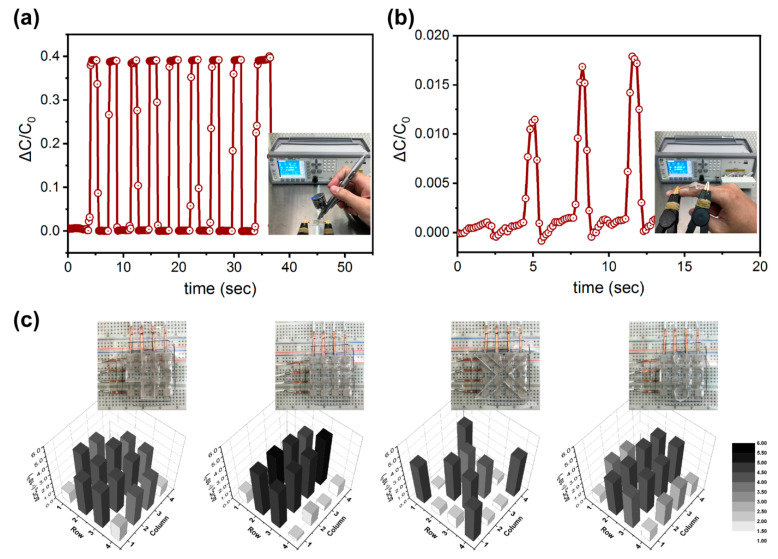
Real-time capacitance variations of the sensor in response to (**a**) air stream pulse and (**b**) finger straightening and bending. (**c**) Spatial pressure distribution and mapping to different math symbols.

In order to explore the potential applications for our AgNW–porous PU capacitive tactile sensors, we monitored the sensing capability to contactless airflow by using an N_2_ spray gun, as shown in Figure 5a. It was found that the discernible signals correlating with the regularly applied airstream pulses could be detected. The relative change in capacitance increased immediately when the airstream was applied; it returned to the initial value rapidly when the airstream was paused. Further, we mounted the device on the proximal interphalangeal (PIP) joint of a volunteer’s index finger, which is an important hinge joint used during gripping movements. The PIP joint that bends and straightens uniaxially with little to no side-to-side movement can change the force applied onto the installed dielectric composite film. We observed that the relative capacitance change varied accordingly (Figure 5b) as the joint moved to different bending angles (~30°, 45°, or 60°). Each time, when the finger was fully straightened (~0°), the value then recovered to its initial state. In addition, a prototype sensor array with 4 × 4 pixels was fabricated to demonstrate the material’s potential as artificial electronic skin. The area of each sensing pixel was 5 mm × 5 mm, with an interval of 5 mm. Silicone rubber film was cut into different math symbols (+, −, ×, and ÷) and placed on the sensor array. Accordingly, the spatial pressure distributions for each loaded symbol are presented in Figure 5c, respectively. The output signals are legible to read and match all the symbols very well, which can reveal great potential for use in human–machine interface applications.

**Figure 6:**
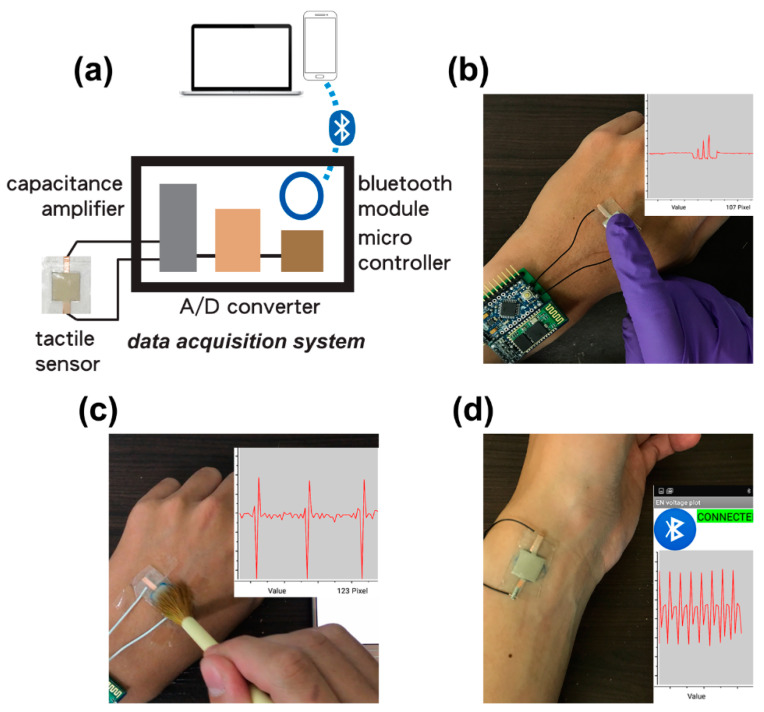
(**a**) Schematic illustration of the self-designed data acquisition (DAQ) system with wireless Bluetooth module. Wireless detection test based on a fabricated capacitive tactile sensor with DAQ system for (**b**) finger tapping, (**c**) brush writing, and (**d**) artery pulse.

Figure 6 presents the wireless application for monitoring human physiological signals. The fabricated AgNW–porous PU capacitive tactile sensor was integrated with a home-built data acquisition (DAQ) circuit board which contained a capacitance amplifier, an A/D converter, a microcontroller, a Bluetooth module, and an Li-ion battery (Figure 6a). The real-time tactile signals generated by physiological movements were collected from the connected sensor and wirelessly transmitted to a mobile phone (or a laptop) through the Bluetooth. For instance, the AgNW–porous PU sensor with DAQ board was affixed to a volunteer’s hand using adhesive tape (Figure 6b), and dynamic signals responding to rapid finger tapping movements could be wirelessly monitored. Additionally, the same setting could detect and convert the force given by sequential brush writing (Figure 6c). Moreover, we relocated the device inverted onto the area between the wrist bone and the tendon on the thumb side of the volunteer’s wrist in order to take their radial arterial pulse (Figure 6d). The typical and regular pulse wave could be read out successfully, presenting a pulse rate of ~70 beats per minute, with two clearly distinguishable peaks. Among these physiological movements, one may notice distinctive downward signals that are lower than the initial values. We suggest that these reduced capacitance signals probably originate from the disturbance of the fringing electric field between the top electrode of the capacitive sensor and a grounded conducting medium (in our case, the user’s finger, hand, or wrist) [40,41]. When the user’s finger or hand is just in contact or in proximity with the top surface of the sensor, there is no pressure applied and no geometric deformation. Though not detailed by our study, these findings may indicate that our proposed device can detect small changes in the electric field between the top electrode and the object in proximity or just in contact (no force applied). These results based on various activities can showcase the potential of our wireless and wearable composite tactile sensors for use in applications related to healthcare electronics and human–machine interfaces.

## 4. Conclusions

In summary, we have demonstrated a wearable and biocompatible capacitive tactile sensor based on a composite dielectric material made of porous elastomeric polyurethane and silver nanowire. By choosing the appropriate dimension of cubic-shaped micropores and loading of nanowires into the matrix, the capacitive tactile sensor exhibits a remarked sensitivity of 0.23 kPa^−1^ and response time of 100 ms, and remains active over a broad range, from as low as 0.3 Pa up to 20 kPa, which can be attributed to the massive change in dielectric deformation and the large variation in relative dielectric permittivity under pressure. All these features, along with remarkable flexibility, conformability, and stability, are very much key to human activity detection, such as contactless airflow, finger bending, and spatial pressure distribution. Additionally, the proposed capacitive tactile sensor integrated with a data acquisition and Bluetooth transmission module can achieve wireless monitoring for radial arterial pulse, making it a promising candidate for artificial skin and healthcare electronics.

## Data Availability

Data is contained within the article and the Supplementary Materials.

## Supplementary Materials

The following supporting information can be downloaded at: https://www.mdpi.com/article/10.3390/polym15183816/s1, Figure S1: TEM characterization for Ag nanowire; Figure S2: Optical images of NaCl cubic crystals; Figure S3: SEM-EDS analysis for a salt crystal-templated porous polyurethane film; Figure S4: Size effect of NaCl-templated cubic pores on pressure sensing behaviors; Figure S5: Proposed sensing mechanisms; Table S1: Summary of recently developed capacitive tactile sensors.
