# Supplementary material for: Wearable Capacitive Tactile Sensor Based on Porous Dielectric Composite of Polyurethane and Silver Nanowire
Source: Polymers (Basel). 2023 Sep 19;15(18):3816. doi: 10.3390/polym15183816 (PMC10535873; doi:10.3390/polym15183816)
Supplement: Supplementary file 1 [file polymers-15-03816-s001.zip › polymers-2615663-supplementary.pdf]

polymers

MDPI

Supplementary Materials: Wearable Capacitive Tactile Sensor Based on Porous Dielectric Composite of Polyurethane and Silver Nanowire

Gen-Wen Hsieh, Chih-Yang Chien

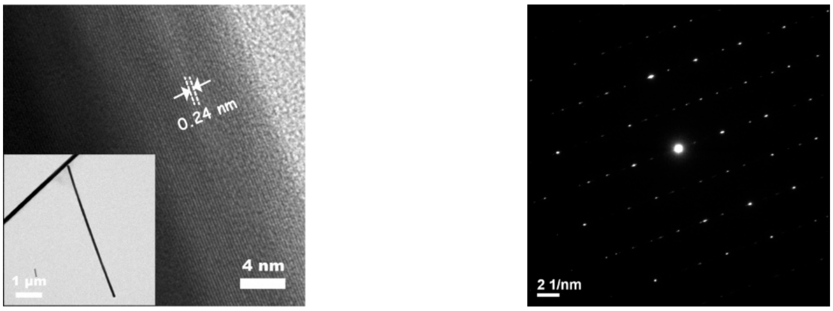

(a) (b)

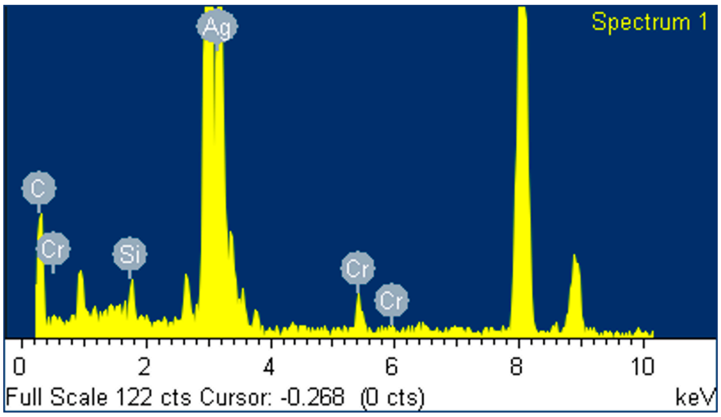

Element	Weight%	Atomic%
C K	3.38	23.21
Si K	0.86	2.54
Cr K	1.39	2.20
Ag L	94.36	72.06
Total	100.00	100.00

(c)

Figure S1. (a) High resolution TEM images and **(b)** a selected area electron diffraction pattern of an as-prepared Ag nanowire, indicating (0002) lattice fringes with interplanar spacings of ~2.4 Å. **(c)** TEM-EDX analysis for a randomly selected Ag nanowire on a carbon-coated TEM copper grid.

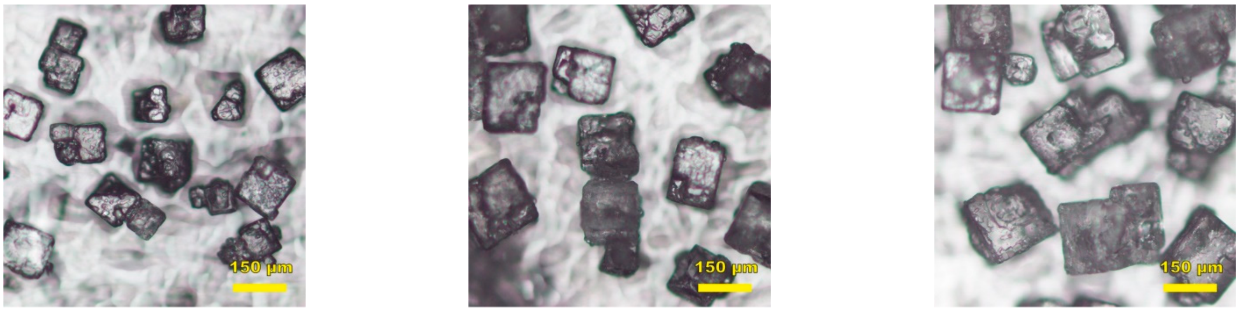

(a) (b) (c)

Figure S2. Optical images of the employed NaCl cubic crystals with lateral size in the range of **(a)** 100–150 μm, **(b)** 150–200 μm, and **(c)** 200–250 μm, respectively.

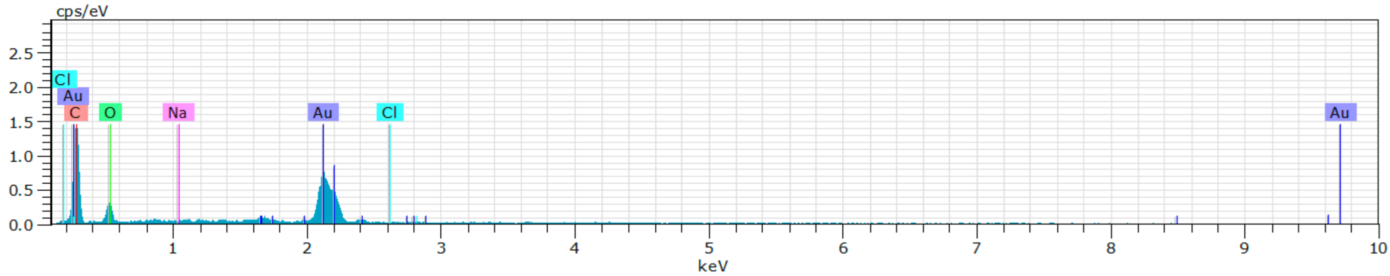

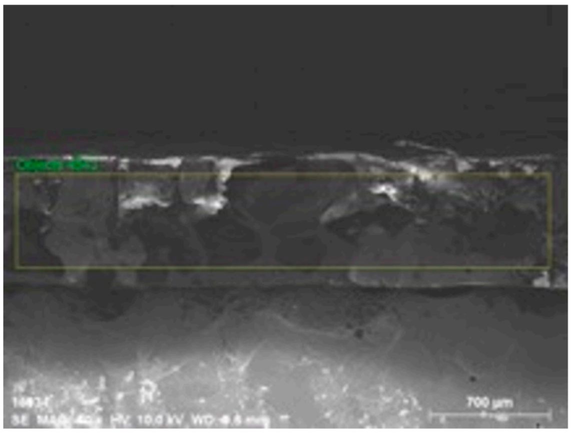

Spectrum: Objects 4543

El	AN	Series	unn. C [wt.%]	norm. C [wt.%]	Atom. C [at.%]	Error (1 Sigma) [wt.%]
C	6	K-series	18.04	30.03	76.35	3.00
O	8	K-series	4.38	7.30	13.93	1.06
Na	11	K-series	0.00	0.00	0.00	0.00
Cl	17	K-series	0.00	0.00	0.00	0.00
Au	79	M-series	37.66	62.68	9.72	1.58
		Total:	60.08	100.00	100.00	

Figure S3. SEM-EDS analysis for a salt crystal-templated porous polyurethane film, confirming no element of Na or Cl remained inside.

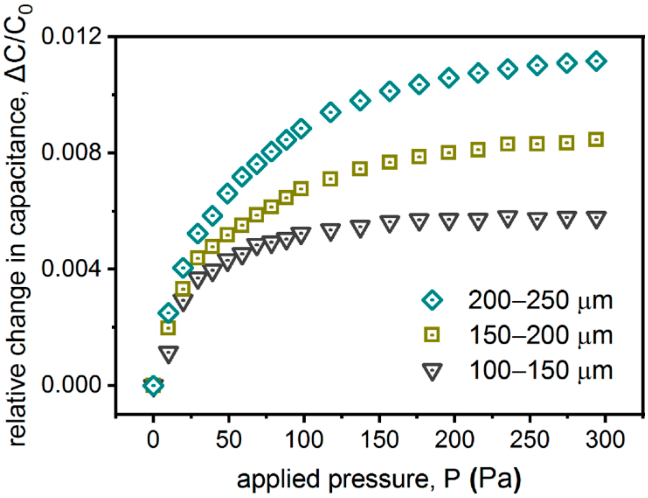

Figure S4. Measured relative capacitance changes as a function of applied pressure for the porous PU capacitive tactile sensors with different lateral sizes of NaCl-templated cubic pores: 100–150, 150–200, and 200–250 µm, respectively.

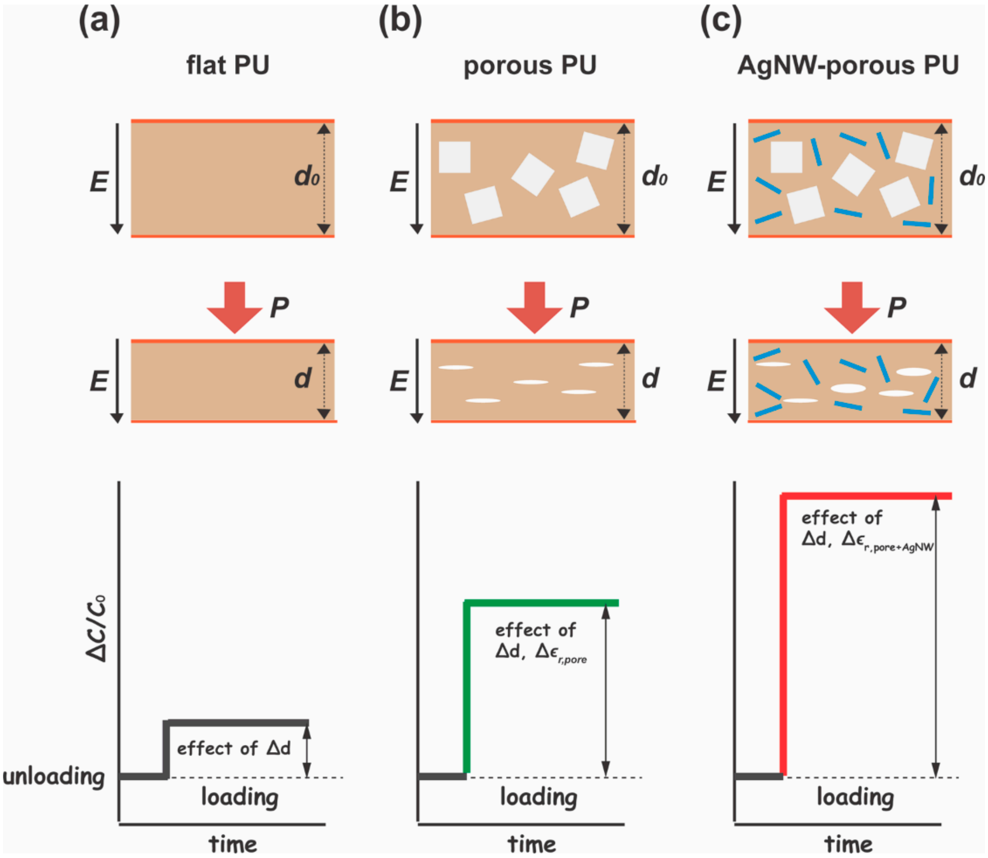

Figure S5. Proposed sensing mechanisms with graphical capacitance changes for the capacitors containing (a) flat PU, (b) porous PU, and (c) AgNW–porous PU.

Table S1. Comparison of the Ag nanowire–porous polyurethane device with other recently developed capacitive tactile sensors.

dielectric	electrode/substrate	max sensitivity (kPa^{-1})	limit of detection (Pa)	response time (ms)	operation range	ref.
PDMS/Ecoflex	CNT/PDMS	0.23	50	125	50 Pa–900 kPa	[24]
pyramidal PDMS	ITO/PET	0.55 (< 2 kPa)	3.0	<<1 s	30 Pa–20 kPa	[2]
micropillar PDMS	Au/PET	0.42 (< 1.5 kPa)	1.0	70	1 Pa–13 kPa	[14]
porous Ecoflex	CNT/Ecoflex	0.601 (< 5 kPa)	0.1–0.2	–	0.1 Pa–130 kPa	[26]
stainless-steel meshed TPU	conductive fabrics	0.182	15	78	15 Pa–40 kPa	[39]
porous pyramid PDMS	ITO/PET	44.5 (< 100 Pa)	0.14	50	0.1 Pa–130 kPa	[27]
ZnO nanowire−PDMS	CNT/PET	6.86×10^{-4}	20	0.9	20 Pa–250 Pa	[21]
ZnO tetrapod−PDMS	PEDTO:PSS	2.55	1.0	180	1 Pa–600 Pa	[22]
parylene/PU nanomesh	Au nanomesh	0.141 (< 1 kPa)	–	190	Up to 100 kPa	[3]
carbon black–porous PDMS/PDMS	CNT/PDMS	35 (< 200 Pa)	9.0	–	9 Pa–12 kPa	[32]
BTO/PVDF fiber + double PDMS microcylinders	Graphene/PI	5	0.11	25	7.5 Pa–40 kPa	[37]
CNT−porous PDMS + CNT−sandpapered PDMS	Cu/PET	1.17	5.0	30	5 Pa–100 kPa	[38]
**Ag nanowire–porous PU**	**Ag nanowire/PET**	**0.23 (< 30 Pa)**	**0.3 Pa**	**100**	**0.3 Pa–20 kPa**	**this work**
